# A Signature of Exaggerated Adipose Tissue Dysfunction in Type 2 Diabetes Is Linked to Low Plasma Adiponectin and Increased Transcriptional Activation of Proteasomal Degradation in Muscle

**DOI:** 10.3390/cells11132005

**Published:** 2022-06-23

**Authors:** Rugivan Sabaratnam, Vibe Skov, Søren K. Paulsen, Stine Juhl, Rikke Kruse, Thea Hansen, Cecilie Halkier, Jonas M. Kristensen, Birgitte F. Vind, Bjørn Richelsen, Steen Knudsen, Jesper Dahlgaard, Henning Beck-Nielsen, Torben A. Kruse, Kurt Højlund

**Affiliations:** 1 Steno Diabetes Center Odense, Odense University Hospital, DK-5000 Odense C, Denmark; rusabaratnam@health.sdu.dk (R.S.); sjpe1@ucl.dk (S.J.); rkruse@health.sdu.dk (R.K.); jmkristensen@nexs.ku.dk (J.M.K.); birgitte.falbe.vind@rsyd.dk (B.F.V.); beck-nielsen@dadlnet.dk (H.B.-N.); 2 Department of Clinical Research, University of Southern Denmark, DK-5000 Odense C, Denmark; christensen-thea@hotmail.com (T.H.); cecilie_halkier@hotmail.com (C.H.); 3 Oxford Centre for Diabetes, Endocrinology and Metabolism, Radcliffe Department of Medicine, University of Oxford, Oxford OX3 7LE, UK; 4 Department of Hematology, Zealand University Hospital, DK-4000 Roskilde, Denmark; vihs@regionsjaelland.dk; 5 Department of Pathology, Viborg Regional Hospital, DK-8800 Viborg, Denmark; soerpaul@rm.dk; 6 Molecular Physiology Section, Department of Nutrition, Exercise and Sports, University of Copenhagen, DK-2100 Copenhagen, Denmark; 7 Steno Diabetes Center Aarhus, Aarhus University Hospital, DK-8200 Aarhus N, Denmark; bjoern.richelsen@aarhus.rm.dk; 8 Allarity Therapeutics Europe, DK-2970 Hørsholm, Denmark; investorrelations@allarity.com; 9 Program for Mind and Body in Mental Health, Research Centre for Health and Welfare Technology, VIA University College, DK-8200 Aarhus, Denmark; jesd@via.dk; 10 Department of Clinical Medicine, Aarhus University, DK-8200 Aarhus, Denmark; 11 Department of Clinical Genetics, Odense University Hospital, DK-5000 Odense C, Denmark; torben.kruse@rsyd.dk

**Keywords:** type 2 diabetes, obesity, adipose tissue dysfunction, skeletal muscle, transcriptomics

## Abstract

Insulin resistance in skeletal muscle in type 2 diabetes (T2D) is characterized by more pronounced metabolic and molecular defects than in obesity *per se*. There is increasing evidence that adipose tissue dysfunction contributes to obesity-induced insulin resistance in skeletal muscle. Here, we used an unbiased approach to examine if adipose tissue dysfunction is exaggerated in T2D and linked to diabetes-related mechanisms of insulin resistance in skeletal muscle. Transcriptional profiling and biological pathways analysis were performed in subcutaneous adipose tissue (SAT) and skeletal muscle biopsies from 17 patients with T2D and 19 glucose-tolerant, age and weight-matched obese controls. Findings were validated by qRT-PCR and western blotting of selected genes and proteins. Patients with T2D were more insulin resistant and had lower plasma adiponectin than obese controls. Transcriptional profiling showed downregulation of genes involved in mitochondrial oxidative phosphorylation and the tricarboxylic-acid cycle and increased expression of extracellular matrix (ECM) genes in SAT in T2D, whereas genes involved in proteasomal degradation were upregulated in the skeletal muscle in T2D. qRT-PCR confirmed most of these findings and showed lower expression of adiponectin in SAT and higher expression of myostatin in muscle in T2D. Interestingly, muscle expression of proteasomal genes correlated positively with SAT expression of ECM genes but inversely with the expression of *ADIPOQ* in SAT and plasma adiponectin. Protein content of proteasomal subunits and major ubiquitin ligases were unaltered in the skeletal muscle of patients with T2D. A transcriptional signature of exaggerated adipose tissue dysfunction in T2D, compared with obesity alone, is linked to low plasma adiponectin and increased transcriptional activation of proteasomal degradation in skeletal muscle.

## 1. Introduction

Type 2 diabetes (T2D) is a multifactorial disease characterized by insulin resistance in adipose tissue, skeletal muscle and liver and β-cell dysfunction leading to hyperglycaemia. Skeletal muscle is the major site of insulin-stimulated glucose disposal, and, hence, a predominant site of insulin resistance in T2D [1]. Using both targeted and omics-based approaches, insulin resistance in human skeletal muscle has been linked to several metabolic and molecular defects related to glucose, lipid, and protein metabolism [2,3,4,5,6,7,8], which are often more pronounced in patients with T2D compared with obesity *per se* [1].

White adipose tissue is an important endocrine organ, which communicates with the rest of the body through the production and secretion of multiple adipokines, contributing to metabolic homeostasis including regulation of insulin sensitivity [9]. Transcriptional profiling as well as targeted studies of genes and proteins in subcutaneous adipose tissue (SAT) have provided evidence of multiple abnormalities, which are collectively termed adipose tissue dysfunction in individuals with obesity-induced insulin resistance [10,11]. Thus, adipose tissue dysfunction in obesity in humans is characterized by adipocyte hypertrophy associated with enhanced extracellular matrix (ECM), focal adhesion and fibrosis, increased macrophage infiltration, and increased release of pro-inflammatory adipokines, while markers of mitochondrial oxidative phosphorylation and secretion of adiponectin are reduced [9,10,11,12]. These alterations are associated with impaired adipose tissue expandability, which together with impaired inhibition of lipolysis causes fat overflow and ectopic-lipid deposition in metabolic tissues, such as the liver and muscle, contributing to insulin resistance and an increased risk of T2D [9]. Moreover, an increased secretion of several pro-inflammatory adipokines and a reduced secretion of adiponectin from SAT are thought to contribute to muscle insulin resistance [13]. Accordingly, SAT expression and circulating levels of adiponectin are reduced in obesity and other insulin-resistant conditions [14,15,16], showing a strong inverse relationship with insulin action on substrate metabolism and insulin signalling in skeletal muscle [16,17]. Taken together, previous studies support the hypothesis that an abnormal interaction between adipose tissue and skeletal muscle could play an important role in obesity-induced insulin resistance. Although targeted studies have shown reduced markers of mitochondrial function and content in SAT in mice and humans with T2D [18,19], it remains to be clarified if known markers of adipose tissue dysfunction in general are exaggerated in T2D compared with obesity alone, and if this is linked to the mechanisms of insulin resistance in skeletal muscle. Moreover, only a few studies have performed transcriptional profiling of both adipose tissue and skeletal muscle to investigate mechanisms of insulin resistance in humans [20,21]. 

In the present study, we applied microarray-based transcriptional profiling and global pathway analysis of both adipose tissue and skeletal muscle biopsies to identify abnormalities in adipose tissue, which are linked to mechanisms of insulin resistance in skeletal muscle of patients with T2D beyond the alterations induced by obesity alone.

## 2. Methods

### 2.1. Study Subjects

Seventeen obese patients with T2D and nineteen 19 non-diabetic control individuals matched according to gender, age, and BMI were included in the study (Table 1). Patients with T2D were treated either by diet alone or diet in combination with metformin, insulin, metformin and insulin, or rosiglitazone and sulfonylurea. Oral anti-diabetics were withdrawn one week prior to the study together with anti-hypertensive and lipid-lowering drugs. Long-acting insulin was withdrawn one day before and rapid-acting insulin the night before the study. Patients with T2D were GAD65 antibody negative and without signs of diabetic retinopathy, nephropathy, neuropathy, or macrovascular complications. All women were postmenopausal. The obese control individuals had normal glucose tolerance, no family history of diabetes, and none were treated with drugs known to affect glucose metabolism. All study participants had normal results on screening blood tests of haematology and hepatic and renal function and were instructed to refrain from strenuous physical activity for a period of 48 h before the experiment. Informed consent was obtained from all individuals before participation. 

### 2.2. Study Design

All study participants were admitted to Odense University Hospital or Aarhus University Hospital, Denmark, after a 10 h overnight fast. Blood samples were drawn in the basal, resting state for measurement of plasma glucose, plasma triglycerides, LDL-cholesterol, HDL-cholesterol, serum insulin, and serum C-peptide as described [22]. Plasma adiponectin was measured using a human-specific high-sensitive ELISA method (B-Bridge International, San Jose, CA, USA). Homeostasis-model assessment of insulin resistance (HOMA-IR), insulin secretion (HOMA-β), and the quantitative insulin-sensitivity check index (QUICKI) were calculated from the overnight-fasting values of plasma glucose and serum insulin as described [23,24]. The reciprocal index of HOMA-IR was also calculated. Muscle biopsies were obtained from the vastus lateralis muscle and adipose tissue biopsies from the abdominal subcutaneous fat using a modified Bergström needle with suction under local anaesthesia (10 mL of lidocaine 2%). Biopsy samples were immediately blotted free of blood and connective tissue, frozen in liquid nitrogen, and stored in −130 °C until analysis. Fat mass and fat-free mass were determined by impedance (Tanita TBF-300 GS, Tanita Corporation, IL, USA).

### 2.3. RNA Extraction and Microarray Preparation

Total RNA was extracted from SAT and skeletal muscle from all participants using the Trizol^TM^ protocol, including an extra phenol-chloroform step as described previously [6]. The quantity of purified RNA was assessed with a NanoDrop^TM^ spectrophotometer ND-8000 (NanoDrop Technologies, Wilmington, DE, USA), while the quality of RNA was assessed using an Agilent 2100 Bioanalyser (Agilent Technologies, Palo Alto, CA, USA). A MessageAmpTM III RNA amplification kit (Ambion, Austin, TX, USA) was used to convert 300 ng of purified RNA to fragmented amplified RNA (aRNA), which was hybridized to Affymetrix HG-U133A 2.0 chips.

### 2.4. Data Processing

Data pre-processing was performed in the statistical programming language R, using the affy package (www.bioconductor.org (accessed on 18 January 2020)). Data was background-corrected with the robust multi-array average-expression measure and normalized using quantile normalization [25]. Gene-expression index calculation was done using model-based index calculation [26]. Only perfectly matched probes were used for data analysis. The regularized *t*-test limma [27] was applied to calculate differences in gene expression between patients and controls, and the Benjamini–Hochberg method using the false-discovery rate (FDR) was used to correct for multiple-hypothesis testing. An uncorrected *p*-value < 0.05 was considered significant.

### 2.5. Biological Pathway Analysis

Database for Annotation, Visualization, and Integrated Discovery (DAVID) Bioinformatics Resources 6.8 [28] and Gene-Set Enrichment Analysis (GSEA) 4.2.2 [29] were applied to identify changes in expression of biological pathways in SAT and skeletal muscle between patients with T2D and obese controls. Using the Functional Annotation Clustering tool in DAVID, we uploaded significantly up- or downregulated genes (*p* < 0.05) and identified regulated clusters of pathways using the annotation categories defined by Biocarta, KEGG and Reactome. Clusters with an enrichment score (ES) > 2.5 were considered significant. Using the MsigDB database in GSEA, we used all gene sets (*n* = 2439) defined in Hallmark gene sets and canonical pathways curated by KEGG, Biocarta, Reactome, and the Pathway-Interaction Database (PID). A gene-set size filter of min 15 and max 500 was applied. All genes in the data set were ranked according to the *t*-test, and 5000 gene permutations were performed to assign the statistical significance of the gene sets. A family-wise error rate (FWER) < 0.05 was considered significant after correction for multiple-hypothesis testing. 

### 2.6. RNA Extraction and cDNA Synthesis

Total RNA from SAT (17 patients with T2D and 19 obese controls) and skeletal muscle (16 patients with T2D and 15 obese individuals) was treated with DNase I (USB, Cleveland, OH, USA) and reverse-transcribed to single-stranded cDNA (Life Technologies/Applied Biosystems, Foster City, CA, USA). Briefly, 5 mg of total RNA was reverse-transcribed to cDNA using a commercially available kit (High Capacity cDNA RT Kit, Life Technologies/Applied Biosystems, Foster City, CA, USA) in accordance with the protocol of the manufacturer including RNase inhibitor.

### 2.7. Quantitative Real-Time PCR (qRT-PCR)

TaqMan^TM^ gene-expression assays (Life Technologies/Applied Biosystems, Foster City, CA, USA) for selected genes in SAT and skeletal muscle (Supplementary Table S1), respectively, were applied to validate gene expression using Applied Biosystems StepOnePlus^TM^ system (Foster City, CA, USA). Expression changes for each SAT gene were normalized to the reference gene *ACTB* (Life Technologies/Applied Biosystems, Foster City, CA, USA), and relative expression levels were calculated using the 2^−ΔΔCT^ method [30]. The muscle transcripts were normalized to three reference genes, *IPO8*, *POLR2A*, and *PPIA*. The data were analysed using qBase^+^ Biogazelle Software version 3.0 (Biogazelle, Zwijnaarde, Belgium) [31] with normalization to the geometric mean of the three reference genes. All gene transcripts were measured in triplicates. 

### 2.8. Muscle Lysates, SDS-PAGE, and Western Blotting

Skeletal-muscle biopsies were freeze-dried, dissected free from blood, visible fat and connective tissues and homogenized (1:80 weight/vol) for 2 × 1 min at 30 Hz using TissueLyser (Qiagen, Copenhagen, Denmark) in ice-cold buffer (pH 7.4) containing: 50 mM HEPES, 150 mM NaCl, 10% glycerol, 1 mM EDTA, 1 mM EGTA, 1% NP-40, 20 mM Na-pyrophosphate, 20 mM β-glycerophosphate, 10 mM NaF, 2 mM PMSF, 10 μg/mL aprotinin, 10 μg/mL leupeptin, 2 mM Na_3_VO_4_ and 3 mM benzamidine [32]. Skeletal muscle homogenates were rotated end-over-end for 1 h at 4 °C and centrifuged at 16,000× *g* at 4 °C for 20 min. Protein concentration was measured by using the bicinchoninic-acid method (Pierce Chemical Co., Rockford, IL, USA). 

Muscle lysates were boiled in Laemmli buffer and separated by SDS-PAGE using self-cast Tris-HCl (8–15%) gels (Bio-Rad, Herlev, Denmark). Following electrophoresis, proteins were transferred to a polyvinylidene fluoride membrane (Immobilon Transfer Membranes; Millipore, Bagsværd, Denmark) by semidry blotting. Membranes were blocked in TBST (10 mM Tris-Base, 150 mM NaCl and 0.25% Tween20) containing either non-fat milk (3%) or BSA (3%) for 1 h at room temperature, washed with TBST, and then incubated with the appropriate primary antibody overnight at 4 °C. Next day, the membranes were incubated with the appropriate secondary antibody for 1 h at room temperature. To visualize the proteins, we used enhanced chemiluminescence (ECL) reagents (Millipore, Burlington, MA, USA) and ChemiDoc XRS+ system (Bio–Rad, Herlev, Denmark). The protein bands were quantified by using Image Lab version 5.2.1 (Bio–Rad, Herlev, Denmark) [32].

### 2.9. Antibodies Used for Western Blotting

Primary antibodies: anti-PSMD1 antibody (Abcam, Cambridge, UK, Ab140682, 1:15,000), anti-PSMB6 antibody (Abcam, Cambridge, UK, Ab3331, 1:1000), anti-PSMB3 antibody (Abcam, Cambridge, UK, Ab88665, 1:1000), anti-PSMA2 (Cell Signalling, MA, USA, #2455, 1:1000), Anti-Atrogin-1 antibody (Abcam, Cambridge, UK, Ab168372, 1:1000), and anti-MURF1 antibody (Abcam, Cambridge, UK, Ab96857, 1:20,000). Secondary antibodies: Rabbit-anti-mouse (DakoCytomation, Glostrup, Denmark P0260, 1:5000) and Goat-anti-rabbit (Jackson-ImmunoResearch Laboratories, West Grove, PA, USA 111-036-045, 1:10,000). 

### 2.10. Statistical Analysis

Statistical analyses were performed using SigmaPlot 13.0 software (Systat Software, San Jose, CA, USA). Differences in clinical and metabolic characteristics and gene expression between groups measured by qRT-PCR or protein abundances were evaluated by unpaired Student’s *t*-test. The relationships between selected variables were examined by calculation of Pearson’s correlation coefficients. Data are presented as means ± SEM and significance was accepted as *p* < 0.05.

## 3. Results

### 3.1. Clinical and Metabolic Characteristics

The two groups were well-matched with respect to age, gender, and degree of obesity as measured by BMI, fat mass, fat-free mass, percentage fat mass, waist or hip circumferences or waist–hip ratio (Table 1). There were no differences between the two groups in the anthropometric measures in either females or males (Supplementary Table S2). Fasting-plasma glucose (*p* < 0.001), HbA_1_C (*p* < 0.001), plasma triacylglycerols (*p* = 0.026) and insulin resistance measured as HOMA-IR (*p* = 0.025) were elevated, whereas plasma adiponectin (*p* = 0.001), indices of insulin sensitivity (QUICKI) (*p* = 0.021), and β-cell function measured as HOMA-β were decreased (*p* = 0.044) in patients with T2D compared with weight-matched controls (Table 1). No differences in cholesterols or liver enzymes were observed between the two groups. 

### 3.2. Effects of T2D on SAT and Muscle Transcripts

Using GSEA, 18 gene sets were significantly downregulated in SAT of patients with T2D (FWER < 0.05). The majority of these gene sets represented mitochondrial oxidative phosphorylation (OXPHOS), the tricarboxylic acid (TCA) cycle, fatty-acid metabolism and branched-chain amino-acid catabolism. Correspondingly, DAVID demonstrated significantly downregulated clusters of pathways (ES > 2.5) represented by genes involved in OXPHOS (Cluster 1) and the TCA cycle (Cluster 3) as well as proteasomal degradation (Cluster 2) in SAT of patients with T2D (Table 2 and Supplementary Material S1 and S2). 

Using GSEA, 25 gene sets were significantly upregulated in SAT of patients with T2D (FWER < 0.05). The majority of these gene sets represented the ECM organization and the complement system. Correspondingly, DAVID demonstrated significantly upregulated clusters of pathways (ES > 2.5), all represented by genes involved in ECM organization, in particular focal adhesion, and ECM-receptor interaction in SAT of patients with T2D (Table 3 and Supplementary Material S1 and S2). Based on consistent findings in GSEA and DAVID, differentially regulated SAT genes representing ECM, OXPHOS, and the TCA cycle were chosen for validation.

Using GSEA, three gene sets were significantly upregulated in the muscle of patients with T2D (FWER < 0.05). These gene sets were all, to a major extent, represented by genes that involved proteasomal degradation (Table 4 and Supplementary Material S3). Correspondingly, DAVID revealed a significantly upregulated cluster (Cluster 1) of pathways (ES > 2.5), all represented by genes involved in ubiquitination and proteasomal degradation (Supplementary Material S4). Neither GSEA nor DAVID identified significantly downregulated gene sets or clusters of pathways in muscle of patients with T2D (Supplementary Material S3 and S4). Based on these results, dysregulated muscle genes involved in proteasomal degradation were chosen for further studies.

### 3.3. Reduced Expression of OXPHOS Genes and ADIPOQ in SAT in T2D

**Figure 1:**
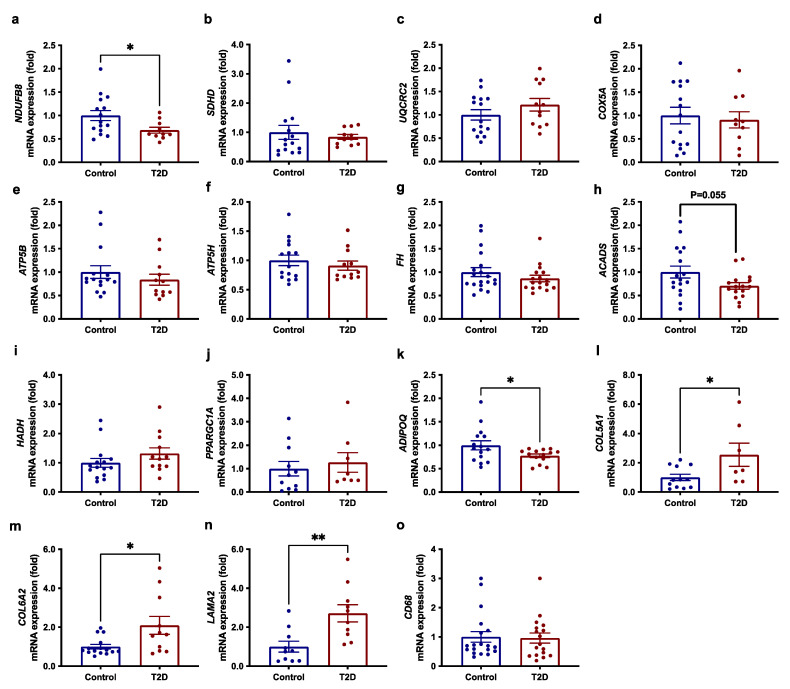
mRNA expression of genes involved in (**a**–**f**) oxidative phosphorylation (*NDUFB8*, *SDHD*, *UQCRC2*, *COX5A*, *ATP5B, ATP5H*), (**g**–**i**) the TCA cycle and fatty-acid oxidation (*FH*, *ACADS,* and *HADH*), (**j**) transcriptional regulation of mitochondrial biogenesis (*PPARGC1A*) and (**k**) adiponectin (*ADIPOQ*) as well as genes involved in (**l**–**n**) extracellular matrix (*COL5A1*, *COL6A2, LAMA2*) and (**o**) macrophage infiltration (*CD68*) in SAT of patients with T2D (*n* = 10–17) vs. obese controls (*n* = 15–19) determined by qRT-PCR. Data are means ± SEM. * *p* < 0.05 and ** *p* < 0.005 vs. obese controls.

Using qRT-PCR, we examined the transcript levels of selected genes from the five respiratory complexes (I-V), TCA cycle, and fatty-acid oxidation, which were found to be downregulated in SAT of patients with T2D in the microarray analysis (*p* < 0.01) as well as the major transcriptional regulator of mitochondrial biogenesis, *PPARGC1A*. We confirmed that the expression of *NDUFB8* (complex I) was downregulated (*p* = 0.039) in SAT of patients with T2D compared with obese controls, whereas expression of genes in the respiratory complexes II-V (*SDHD, UQCRC2, COX5A*, *ATP5B,* and *ATP5H*), the TCA cycle (*FH*) and fatty-acid oxidation (*HADH* and *ACADS*) as well as *PPARGC1A* were not significantly downregulated in SAT of patients with T2D (Figure 1a–j). Consistent with the lower plasma adiponectin, transcript levels of *ADIPOQ* in SAT were reduced (*p* = 0.047) in patients with T2D compared with obese controls (Figure 1k). 

### 3.4. Increased Expression of ECM Genes in SAT in T2D

In agreement with the results of the microarray analysis, we show increased expression of selected ECM genes using qRT-PCR (Figure 1l–n). Thus, the expression of *COL5A1* (*p* = 0.029), *COL6A2* (*p* = 0.014), and *LAMA2* (*p* = 0.004) was increased in SAT of patients with T2D compared with obese controls. In obesity, a link between enhanced ECM, fibrosis, and adipose tissue macrophage infiltration has been reported [9]. However, the transcript levels of a marker of macrophage infiltration, *CD68*, were not increased in patients with T2D compared with obese controls (Figure 1o). 

### 3.5. Increased Expression of Proteasomal-Degradation Genes in Muscle T2D

**Figure 2:**
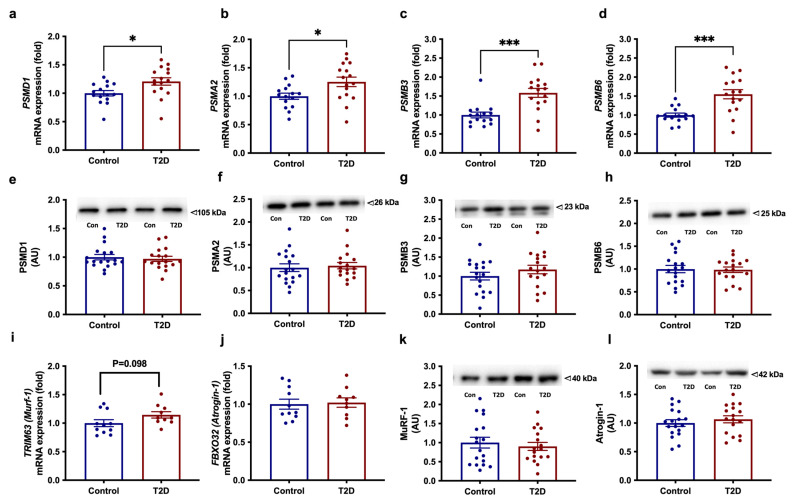
mRNA expression and protein abundances of genes involved in proteasomal degradation; (**a**–**h**) the proteasomal subunits PSMA2, PSMB3, PSMB6, and PSMD and (**i**–**l**) the muscle-specific E3 ubiquitin ligases TRIM63 (encoding MuRF-1) and FBXO32 (encoding Atrogin-1) in skeletal muscle of patients with T2D (*n* =10–17) vs. obese controls (*n* = 10–19). Data are means ± SEM. * *p* < 0.05 and *** *p* < 0.001 vs. obese controls.

Pathway analysis of the microarray data showed enhanced expression of proteasomal degradation genes in skeletal muscle of patients with T2D. Using qRT-PCR, we confirmed that the expression of *PSMD1* (*p* = 0.017), *PSMA2* (*p* = 0.018), *PSMB3* (*p* = 0.0003) and *PSMB6* (*p* = 0.0003) was markedly increased in muscle of patients with T2D compared with obese individuals (Figure 2a–d). We next examined the protein abundance of PSMD1, PSMA2, PSMB3, and PSMB6 but could not demonstrate any differences between the two groups (Figure 2e–h). 

Next, we examined expression of other key genes known to regulate muscle mass through proteasomal degradation, including members of the ubiquitin–proteasome system (UPS) and the myostatin-signalling pathway as well as downstream myogenic factors and regulators of muscle-fibre-type composition. Expression of the muscle-specific E3 ubiquitin ligase *TRIM63* (encoding MuRF-1) tended to be increased (*p* = 0.098) in patients with T2D compared to obese controls, whereas the expression of another muscle-specific E3 ubiquitin ligase, *FBXO32* (encoding Atrogin-1), and its upstream regulator *FOXO3* did not differ between the groups (Figure 2i–j and Supplementary Table S3). Moreover, we observed no differences in protein abundance of Atrogin-1 and MuRF-1 between the two groups (Figure 2k–l). Expression of *MSTN* (or *GDF8,* encoding the myokine, myostatin) (*p* = 0.005) and its receptor, *ACVR2B* (*p* = 0.048) was upregulated in muscle of patients with T2D compared with obese individuals (Supplementary Table S3). No differences in the muscle expression of the myostatin-inhibitory factor *SMAD7* or the myogenic factors *MEF2C, MYOG, MYF5*, and *MYF6* were found between groups, except for a tendency toward a reduced expression of *MYOD1* (*p* = 0.064) in patients with T2D. Muscle expression of *MYH7* (encoding MHC I, slow-oxidative type 1 fibres) and *MYH2* (encoding MHC IIa, fast-oxidative type 2a fibres) did not differ between groups, but the expression of *MYH1* (encoding MHC IIx, glycolytic type 2x fibres) tended (*p* = 0.061) to be increased in patients with T2D (Supplementary Table S3). Among regulators of fibre-type-switching, muscle expression of *PPP3CA* (encoding a catalytic subunit in calcineurin) was increased (*p* = 0.0004) in T2D, whereas the expression of *PPP3CB* and *MYOZ1* were similar in the two groups. 

### 3.6. Expression of Muscle Transcripts Involved in Substrate Metabolism

Several previous studies have reported reduced markers of mitochondrial oxidative phosphorylation, TCA cycle, and lipid metabolism, and increased markers of glycolysis in skeletal muscle of individuals with T2D, obesity, and other insulin-resistant conditions compared with lean, healthy individuals [4,5,6,7,8]. However, using qRT-PCR, we found no significant differences in the muscle expression of selected genes involved in oxidative phosphorylation (*NDUFS1*, *UQCRC1, COX5B*, *ATP5)*, TCA cycle (*SDHA*, *CS*), lipid metabolism (*CPT1B*, *ACADVL, ETFA*), glycolysis (*PGAM2*), or regulators of these processes (*ADIPOR1*, *ADIPOR2*, *CD36*, *PRKAA2*, *PDK4*, *PPARGC1A*) between the groups (Supplementary Table S3). Only the expression of *PRKAG3* (encoding AMPK γ3 subunit) (*p* = 0.063) and *HADH* (*p* = 0.075) tended to be increased in patients with T2D compared with obese controls (Supplementary Table S3). 

### 3.7. Expression of Pro-Inflammatory Adipokines and Putative Myokines

Adipokines or myokines are believed to play key roles in mediating the inter-organ crosstalk between adipose tissue and muscle or other tissue. The microarray analysis did not reveal any pathways directly related to adipokines or myokines. We, therefore, explored the microarray dataset to evaluate whether known pro-inflammatory adipokines or putative myokines were differently expressed in SAT or muscle of patients with T2D compared with obese controls (Supplementary Table S4). However, expression of the genes encoding leptin, apolipoprotein E, resistin, IL-6, MCP-1, RBP4, fatty acid binding protein 4, adipsin, visfatin and chemerin in SAT were similar (all FDR > 0.1) in the two groups. In skeletal muscle, the expression of *MSTN* (or *GDF8*, encoding myostatin) were 1.7-fold increased (unadjusted *p* = 0.03) in T2D, whereas the expression of other putative myokines *DCN*, *SPARC*, *IL6, IL10*, *IL15*, *ANGPTL4*, *FGF21*, *FGF2, FSLT1, LIF, CHI3L1, GDF15*, *BDNF*, or *CTGF* did not differ between groups (all FDR > 0.1). The increased expression of *MSTN* was validated by qRT-PCR (Supplementary Table S3). 

### 3.8. Correlation Analyses

**Figure 3:**
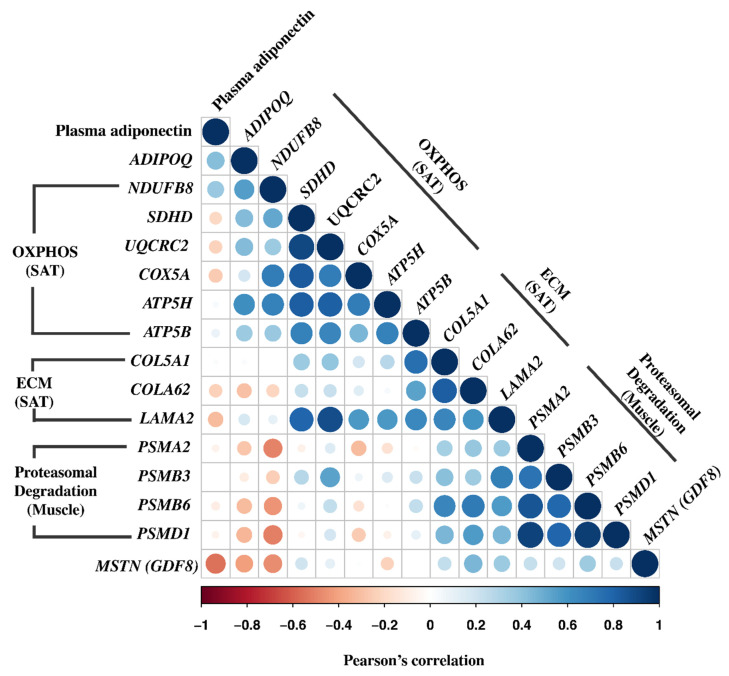
Correlation matrix of gene expression levels of differently regulated SAT and muscle transcripts validated by qRT-PCR and plasma adiponectin in the total cohort (*n* = 36). The intensity of the colour and the size of the dots indicate the strength of the Pearson’s correlation coefficient.

To explore the potential relationship between genes showing differential expression in either SAT or muscle validated by qRT-PCR as well as plasma adiponectin, we performed simple correlation analysis (*n* = 36) in the total cohort of study participants (Figure 3). Expression of *NDUFB8* correlated positively with expression of *ADIPOQ* in SAT (*r* = 0.70; *p* < 0.01) and plasma adiponectin (*r* = 0.63; *p* < 0.01), and correlated inversely with muscle expression of *PSMA2*, *PSMB6*, *PSMD1* (*r* = −0.44 to −0.53; all *p* < 0.05). Moreover, expression of *NDUFB8* was highly co-regulated (*r* = 0.41–0.66; all *p* < 0.05) with expression of the other OXPHOS genes (*SDHD, UQCRC2, COX5A*, *ATP5B, ATP5H*) in SAT. Expression of *ADIPOQ* in SAT correlated positively with plasma adiponectin (*r* = 0.60; *p* < 0.01) and expression of other OXPHOS genes *SDHD, UQCRC2, ATP5B, ATP5H*) in SAT (*r* = 0.44–0.65; all *p* < 0.05), and correlated inversely with muscle expression of *PSMD1* (*r* = −0.38; *p* < 0.05). Interestingly, expression of ECM genes in SAT correlated positively with expression of proteasomal degradation genes in muscle. Thus, *COL5A1* in SAT correlated positively with *PSMB3* and *PSMB6* in muscle (*r* = 0.48–0.63; all *p* < 0.01), *COL6A2* in SAT correlated positively with *PSMB6* in muscle (*r* = 0.65; *p* < 0.01), and *LAMA2* in SAT correlated with *PSMB3* and *PSMB6* in muscle (*r* = 0.45–0.57; all *p* < 0.05). In addition, the muscle expression of all proteasomal degradation genes (*PSMA2*, *PSMB3*, *PSMB6*, *PSMD1*) were strongly co-regulated (*r* = 0.83–0.95; all *p* < 0.01), and they all correlated inversely with plasma adiponectin (*r* = −0.37 to −0.41; all *p* < 0.05). The muscle expression of *MSTN* also correlated inversely with plasma adiponectin (*r* = 0.63; *p* < 0.01), but not with OXPHOS or ECM genes in SAT or proteasomal degradation genes in muscle.

## 4. Discussion

In the present study, we combined transcriptomics and biological pathway analysis to compare the transcriptional profiles of SAT and skeletal muscle between obese patients with T2D and glucose-tolerant obese individuals. We tested the hypothesis that adipose tissue dysfunction is exaggerated in T2D compared with obesity alone and if this is linked to the mechanisms of insulin resistance in skeletal muscle. Using this unbiased approach, we demonstrate increased expression of ECM and focal adhesion genes and decreased expression of genes involved in mitochondrial OXPHOS and the TCA cycle in SAT of patients with T2D compared with obese controls. These changes were accompanied by lower expression of *ADIPOQ* in SAT, reduced plasma adiponectin and increased expression of genes involved in proteasomal degradation in skeletal muscle of patients with T2D compared with obese controls. Interestingly, muscle expression of proteasomal degradation genes correlated positively with SAT expression of ECM genes and inversely with SAT expression of *NDUFB8* and *ADIPOQ* and plasma adiponectin. Overall, our data suggest a transcriptional signature of exaggerated adipose tissue dysfunction, which is linked to increased transcriptional activation of proteasomal degradation in muscle of patients with T2D as compared with obesity alone, and that the latter could be mediated by reduced expression and circulating levels of adiponectin, although a role for other adipokines or myokines cannot be ruled out. 

Previous studies have demonstrated a role for abnormalities in mitochondrial oxidative phosphorylation in adipose tissue dysfunction in obesity-induced insulin resistance. This includes targeted studies showing reduced markers of mitochondrial content and function in SAT of obese (*db*/*db*) mice [33] as well as unbiased, omics-based studies showing impaired mitochondrial biogenesis in individuals with acquired obesity compared with their lean co-twins [12,34] and reduced expression of genes involved in OXPHOS and the TCA cycle in obese, insulin-resistant individuals [11,20,35]. In addition, a few targeted studies have suggested reduced levels of some markers of mitochondrial function and content in SAT in diabetic (*db*/*db*) mice and patients with T2D compared with overweight/obesity alone [18,19,36]. In the present study, we extend these findings. Thus, using an unbiased approach, we provide evidence in a large and well-matched cohort that transcriptional downregulation of genes involved in OXPHOS and TCA cycle in SAT is exaggerated in patients with T2D compared with obesity alone. As noted in other studies [4,5,37], qRT-PCR could not confirm reduced expression of all selected mitochondrial genes in SAT from patients with T2D compared with obese individuals. This lack of consistency is likely explained by the fact that microarray-based transcriptional profiling takes advantage of pathway analysis, which can point out subtle but strongly coordinated changes in pathways [4,5]. Overall, our data support previous targeted studies showing that this component of adipose tissue dysfunction is more severely affected in T2D compared with obesity alone. 

Adipose tissue dysfunction in obesity is characterized by hypertrophic expansion associated with excessive accumulation of ECM components, increased fibrosis, and exaggerated infiltration with pro-inflammatory macrophages, and, hence, a link between these cellular processes has been proposed [9]. ECM remodelling plays an important role in orchestrating the architecture of adipose tissue, especially in SAT expansion (hyperplasia vs. hypertrophy), and enhanced markers of ECM remodelling have been reported in SAT of obese, insulin-resistant individuals [10,38,39]. Interestingly, a moderate weight-loss program in obese individuals caused downregulation of ECM genes in SAT, indicating that this part of adipose tissue dysfunction is at least in part reversible [40]. In the present study, we, for the first time, demonstrate an enhanced expression of genes involved in ECM organization and focal adhesion in SAT from patients with T2D compared with glucose-tolerant, obese individuals. The up-regulation of the ECM genes *COL5A1*, *COL6A2*, and *LAMA2* in T2D was confirmed by qRT-PCR. Our data suggest that increased levels of ECM components in T2D could further limit SAT expandability, which would further increase fat overflow and risk of ectopic-lipid deposition in the liver and skeletal muscle in T2D as compared with obesity alone. However, additional studies are needed to understand if the enhanced expression of ECM genes in T2D vs. obesity alone is a cause or consequence of the development of T2D, and to what extent this component of adipose tissue dysfunction in T2D is reversible. 

Results from previous studies of SAT from obese, insulin-resistant individuals and monozygotic twins discordant for obesity have provided evidence for increased levels of genes involved in the inflammatory pathways, including the complement cascade and macrophage infiltration [11,20,34,39,41,42]. Although GSEA pointed out upregulation of pathways belonging to the complement cascade in SAT of patients with T2D compared with obese controls, these pathways did not show enriched clustering when using DAVID, and, therefore, we did not focus further on inflammatory pathways in this study. Additionally, we did not find any difference in the expression of *CD68* in SAT between patients with T2D and obese controls, suggesting a similar degree of macrophage infiltration. However, based on our results alone, we are unable to rule out that this component of adipose tissue dysfunction in obesity-related insulin resistance is exaggerated in T2D. 

Increased protein degradation and decreased muscle mass in T2D is believed to be a consequence of decreased insulin responsiveness, inflammation, and other factors, which ultimately activates protein-catabolic pathways [43,44]. However, the reported effects of T2D on changes in protein metabolism and muscle mass are not as well-established as in type 1 diabetes [43,44]. A previous transcriptomic study showed upregulation of ubiquitination and protein-degradation pathways in individuals with obesity-induced insulin resistance [20]. In the present study, we extend this finding by showing increased transcriptional activation of pathways involved in ubiquitination and proteasomal degradation in muscle of patients with T2D compared with obese individuals. qRT-PCR analysis confirmed increased muscle expression of several selected proteasome subunits in patients with T2D. However, the protein abundance of these proteasome subunits was unaltered in patients with T2D compared with obese individuals. In line with this, a quantitative proteomics study reported increased protein levels of various 26S and the 20S proteasome subunits in muscle of patients with T2D and obese individuals compared with lean individuals, but with no difference between T2D and obesity [8]. 

In addition to upregulation of proteasome subunits, the pathway (R-HSA-983168) with the highest number of upregulated genes in Cluster 1 of the DAVID analysis of muscle also included several E2 ubiquitin-conjugating enzymes and E3 ubiquitin ligases (Supplementary Material S4), which marks substrate proteins for proteasomal degradation [45]. However, focusing on the two most well-established muscle-specific E3 ubiquitin ligases, we only found a tendency for increased muscle expression of *TRIM63* (encoding MuRF-1) in patients with T2D, whereas the expression of *FBXO32* (encoding Atrogin-1) and its upstream regulator *FOXO3* (encoding Forkhead-box protein O3) did not differ between the groups. As with the proteasome subunits, we found no differences in the protein abundance of Atrogin-1 or MuRF1 between the groups. Myostatin, which is another critical regulator of skeletal muscle mass [46], has been reported to be increased in muscle in obese individuals [47]. Since myostatin signalling along the activin receptor type-IIB (ACTR-IIB) also stimulates proteasomal degradation [48], we measured expression of *MSTN* and its receptor *ACVR2B*, which were both increased in muscle of patients with T2D compared with obese individuals. Downstream of ACTR-IIB, there were no significant differences in the gene expression of myogenic factors or SMAD7. Overall, our findings of increased transcriptional activation of genes involved in ubiquitination and proteasomal degradation and myostatin signalling in muscle of patients with T2D compared with obesity alone were not supported by the abundance of proteins in the ubiquitin-proteasomal system. This could indicate that protein degradation or loss of muscle mass are not enhanced in patients with T2D compared with obesity alone. However, we cannot exclude that the proteasomal activity or turnover of these proteins in muscle are increased in patients with T2D compared with obese individuals. 

Reduced SAT expression and secretion of adiponectin are markers of adipose tissue dysfunction and are strongly linked to both obesity and insulin resistance [15,16]. Circulating adiponectin is even more reduced in patients with T2D [16] and predicts an increased risk of T2D [17,49]. Furthermore, circulating adiponectin shows a strong inverse relationship with insulin action on substrate metabolism and insulin signalling in skeletal muscle [16,17]. Here, we show that both the SAT expression of *ADIPOQ* and circulating levels of adiponectin are further decreased in patients with T2D compared with obese individuals. Studies in mice have provided evidence that low circulating adiponectin levels may stimulate increased muscle protein degradation via activation of the ubiquitin-proteasomal system, suggesting a role for adipose tissue–muscle crosstalk [50,51]. Intriguingly, in the present study, we found that increased muscle expression of proteasomal degradation genes was associated not only with lower SAT expression of *ADIPOQ* and plasma levels of adiponectin but also with other markers of adipose tissue dysfunction, such as increased expression of ECM genes and decreased expression of OXPHOS genes in SAT. These data support studies in mice [33,50,51] and provide correlative evidence suggesting that these components of adipose tissue dysfunction, possibly through reduced secretion of adiponectin, may exert deleterious effects on skeletal muscle protein metabolism, and that these mechanisms might be exaggerated in patients with T2D compared with obesity alone. However, further studies are needed to establish the underlying molecular mechanisms and the possible involvement of additional pro-inflammatory adipokines and even myokines in humans. 

As noted above, several studies using transcriptional profiling have demonstrated reduced expression of OXPHOS genes in skeletal muscle in different insulin-resistant conditions compared with lean, healthy individuals [4,5,6,52]. However, despite higher HOMA-IR, we did not identify downregulation of OXPHOS genes in skeletal muscle of patients with T2D compared with glucose-tolerant, obese individuals either by transcriptional profiling or qRT-PCR analysis. This lack of difference in muscle mRNA levels of OXPHOS genes between patients with T2D and glucose-tolerant, obese individuals has also been reported in an even larger cohort, suggesting that our finding is not caused by the sample size [53]. While changes in gene expression are not always mirrored by changes in protein abundance, the results are in line with previous studies using either proteomics or western blotting, showing a clear reduction in muscle abundance of OXPHOS proteins in patients with T2D compared to lean, healthy individuals, with no or only a smaller reduction compared with non-diabetic, obese individuals [7,8,54]. These findings, however, do not exclude that mitochondrial function is reduced in skeletal muscle of patients with T2D compared with non-diabetic, obese individuals, as shown previously [55,56]. 

Potential limitations of our study include the observational nature of the study, the lack of a group of healthy, lean individuals to point out abnormalities caused by obesity alone, the lack of sufficient tissue material for further validation using histological examinations and additional measures of protein abundance and enzymatic activity, and the lack of assessment of physical-activity level in the study participants. The strengths of our study include the relatively large sample size of well-matched patients with T2D and obese, glucose-tolerant individuals and the inclusion of both men and women, although separate analyses of transcript data for each gender were not carried out. 

In conclusion, our study demonstrates a transcriptional signature of exaggerated adipose tissue dysfunction in patients with T2D compared with obesity alone, and that this is linked to increased transcriptional activation of ubiquitination and proteasomal degradation in skeletal muscle. Correlation analysis supports a possible link between these transcriptional changes and suggests that the accompanying reduced expression and secretion of adiponectin may, at least in part, mediate the changes observed in muscle in patients with T2D. However, further functional studies are needed to explore the association between these abnormalities and understand their potential role in the development of T2D. 

## Figures and Tables

**Table 1:** Clinical and metabolic characteristics.

	Obese Controls	Type 2 Diabetes
Male/female	12/7	10/7
Age (years)	57.1 ± 1.5	56.8 ± 1.5
BMI (kg/m^2^)	30.2 ± 1.1	30.5 ± 0.6
Fat mass (kg)	30.8 ± 2.3	31.6 ± 2.0
Fat-free mass (kg)	59.0 ± 2.6	58.6 ± 3.0
Percentage fat mass (%)	35.2 ± 1.9	34.1 ± 2.0
Waist circumference (cm)	105 ± 3	103 ± 3
Hip circumference (cm)	107 ± 3	106 ± 3
Waist–hip ratio	0.98 ± 0.02	0.98 ± 0.02
Systolic blood pressure (mmHg)	138 ± 3	154 ± 7 *
Diastolic blood pressure (mmHg)	84 ± 2	89 ± 3
Fasting plasma glucose (mmol/L)	5.3 ± 0.1	7.5 ± 0.4 ***
HbA_1_c (%)	5.4 ± 0.1	7.6 ± 0.5 ***
Serum insulin (pmol/L)	63 ± 10	80 ± 12
Serum C-peptide (pmol/L)	932 ± 111	1225 ± 117
Total cholesterol (mmol/L)	5.7 ± 0.2	5.4 ± 0.3
LDL cholesterol (mmol/L)	3.6 ± 0.2	3.1 ± 0.2
HDL cholesterol (mmol/L)	1.5 ± 0.1	1.4 ± 0.1
Plasma triacylglycerol (mmol/L)	1.5 ± 0.1	2.0 ± 0.2 *
Plasma adiponectin (mg/L)	11.2 ± 1.1	6.8 ± 0.5 ***
Alanine aminotransferase (U/L)	36 ± 9	40 ± 6
Alkaline phosphatase (U/L)	70 ± 4	76 ± 4
HOMA-IR	2.5 ± 0.43	4.5 ± 0.7 *
QUICKI	0.346 ± 0.007	0.319 ± 0.008 *
1/HOMA-IR	0.58 ± 0.07	0.36 ± 0.07 *
HOMA-β	116 ± 17	73 ± 10 *

Study participants were studied after an overnight fast. Data represent means ± SEM. * *p* < 0.05 and *** *p* < 0.001 vs. obese individuals. QUICKI; Quantitative insulin-sensitivity check index, HOMA-IR; Homeostasis-model assessment of insulin resistance and HOMA-β; Homeostasis-model assessment of β-cell function.

**Table 2:** Downregulated gene sets in SAT of patients with T2D analysed with GSEA.

Name	Database	Size	ES	NES	NOM *p*-Value	FDR q-Value	FWER *p*-Value
The citric acid TCA cycle and respiratory electron transport	R	145	0.59	2.81	<0.0001	<0.0001	<0.0001
Respiratory electron transport	R	79	0.64	2.72	<0.0001	<0.0001	<0.0001
Respiratory electron transport ATP synthesis by chemiosmotic coupling and heat production by uncoupling proteins	R	99	0.61	2.68	<0.0001	<0.0001	<0.0001
Oxidative phosphorylation	H	200	0.54	2.66	<0.0001	<0.0001	<0.0001
Striated muscle contraction	R	35	0.71	2.53	<0.0001	<0.0001	<0.0001
Complex I biogenesis	R	43	0.65	2.47	<0.0001	<0.0001	<0.0001
Oxidative phosphorylation	K	104	0.51	2.29	<0.0001	0.0002	0.0014
Parkinson’s disease	K	104	0.51	2.28	<0.0001	0.0002	0.0018
Mitochondrial translation	R	57	0.56	2.27	<0.0001	0.0002	0.0018
Huntington’s disease	K	154	0.47	2.22	<0.0001	0.0006	0.0062
Pyruvate metabolism and citric acid TCA cycle	R	50	0.57	2.21	<0.0001	0.0006	0.0078
Mitochondrial protein import	R	54	0.56	2.20	<0.0001	0.0006	0.0084
Mitochondrial fatty acid beta oxidation	R	27	0.65	2.17	<0.0001	0.0009	0.0126
Citrate cycle TCA cycle	K	30	0.63	2.16	0.0004	0.0009	0.0144
Valine leucine and isoleucine degradation	K	41	0.57	2.12	<0.0001	0.0015	0.0240
Cristae formation	R	26	0.63	2.12	<0.0001	0.0014	0.0244
Fatty acid metabolism	K	39	0.57	2.12	<0.0001	0.0014	0.0252
Biosynthesis of unsaturated fatty acids	K	20	0.68	2.10	0.0004	0.0019	0.0364

Shown are the significantly downregulated gene sets in SAT of patients with T2D compared to obese controls (FWER < 0.05). Ranking of the gene sets was done using GSEA 4.2.2. Pathway databases: H, Hallmark; K, KEGG; R, Reactome; ES, enrichment score; NES, normalized enrichment score; NOM, nominal; FDR, false-discovery rate; FWER, family-wise error rate.

**Table 3:** Upregulated gene sets in SAT of patients with T2D analysed with GSEA.

Name	Database	Size	ES	NES	NOM *p*-Value	FDR q-Value	FWER *p*-Value
Epithelial mesenchymal transition	H	196	−0.54	−2.59	<0.0001	<0.0001	<0.0001
Initial triggering of complement	R	33	−0.71	−2.43	<0.0001	<0.0001	<0.0001
CD22 mediated BCR regulation	R	20	−0.77	−2.37	<0.0001	0.0001	0.0002
Extracellular matrix organization	R	266	−0.47	−2.32	<0.0001	0.0001	0.0004
Scavenging of heme from plasma	R	26	−0.71	−2.31	<0.0001	0.0001	0.0004
Creation of C4 and C2 activators	R	27	−0.70	−2.31	<0.0001	0.0001	0.0004
Molecules associated with elastic fibres	R	36	−0.64	−2.27	<0.0001	0.0002	0.0012
Elastic fibre formation	R	41	−0.62	−2.27	<0.0001	0.0002	0.0014
Complement cascade	R	66	−0.56	−2.26	<0.0001	0.0002	0.0020
FCERI mediated MAPK activation	R	46	−0.60	−2.24	<0.0001	0.0003	0.0024
Assembly of collagen fibrils and other multimeric structures	R	54	−0.58	−2.24	<0.0001	0.0002	0.0024
Antigen activates B-cell receptor BCR leading to generation of second messengers	R	42	−0.61	−2.23	<0.0001	0.0003	0.0030
ECM proteoglycans	R	73	−0.54	−2.22	<0.0001	0.0004	0.0048
FCGR activation	R	26	−0.68	−2.21	<0.0001	0.0004	0.0052
Collagen formation	R	74	−0.54	−2.21	<0.0001	0.0004	0.0052
Degradation of the extracellular matrix	R	121	−0.48	−2.17	<0.0001	0.0006	0.0086
Collagen degradation	R	57	−0.54	−2.13	<0.0001	0.0011	0.0174
MET activates PTK2 signaling	R	26	−0.65	−2.13	<0.0001	0.0011	0.0184
Chondroitin sulfate dermatan sulfate metabolism	R	40	−0.59	−2.13	<0.0001	0.0011	0.0188
Syndecan 4 pathway	P	30	−0.63	−2.13	<0.0001	0.0011	0.0192
Collagen biosynthesis and modifying enzymes	R	53	−0.55	−2.12	<0.0001	0.0011	0.0216
Syndecan 1 pathway	P	45	−0.57	−2.12	<0.0001	0.0011	0.0218
Integrin cell surface interactions	R	79	−0.51	−2.12	<0.0001	0.0010	0.0218
Parasite infection	R	71	−0.52	−2.12	<0.0001	0.0010	0.0220
Integrin1 pathway	P	61	−0.53	−2.11	<0.0001	0.0012	0.0262

Shown are the significantly upregulated gene sets in SAT of patients with T2D compared to obese controls (FWER < 0.05). Ranking of the gene sets was done using GSEA 4.2.2. Pathway databases: H, Hallmark; R, Reactome; P, PID; ES, enrichment score; NES, normalized enrichment score; NOM, nominal; FDR, false-discovery rate; FWER, family-wise error rate.

**Table 4:** Upregulated gene sets in skeletal muscle of patients with T2D analysed with GSEA.

Name	Database	Size	ES	NES	Nom *p*-Value	FDR q-Value	FWER *p*-Value
Proteasome pathway	B	19	−0.67	−2.40	<0.0001	0.0081	0.0030
Regulation of HMOX1 expression and activity	R	61	−0.44	−2.16	<0.0001	0.0632	0.0474
Proteasome	K	42	−0.48	−2.15	<0.0001	0.0443	0.0498

Shown are the significantly upregulated gene sets in skeletal muscle of patients with type 2 diabetes compared to obese controls (FWER < 0.05). Ranking of the gene sets was done using GSEA 4.2.2. Pathway databases: B, Biocarta; K, KEGG; R, Reactome; ES, enrichment score; NES, normalized enrichment score; NOM, nominal; FDR, false-discovery rate; FWER, family-wise error rate.

## Data Availability

The data that support the findings of this study are available from the corresponding author upon reasonable request.

## Supplementary Materials

The following supporting information can be downloaded at: https://www.mdpi.com/article/10.3390/cells11132005/s1, File S1: Ranking of the gene sets using Gene Set Enrichment Analysis (GSEA) version 4.2.2. The list shows significantly downregulated gene sets in SAT of patients with T2D compared to obese controls; File S2: Clusters of pathways downregulated in subcutaneous adipose tissue (SAT) of patients with T2D; File S3: Ranking of the gene sets using Gene Set Enrichment Analysis (GSEA) version 4.2.2. The list shows significantly upregulated gene sets in skeletal muscle of patients with T2D compared to obese controls; File S4: Clusters of pathways upregulated in skeletal muscle of patients with T2D; Table S1: TaqMan assays for the selected genes validated with qRT-PCR; Table S2: Anthropometric characteristics of males and females; Tables S3: mRNA expression of genes involved in regulation of muscle mass and substrate metabolism; Table S4: Expression of pro-inflammatory adipokines and putative myokines.

## Abbreviations

BMI—Body mass index; DAVID—Database for Annotation, Visualization, and Integrated Discovery; ECM—Extracellular matrix; FDR—False-discovery rate; FWER—Family-wise error rate; GSEA—Gene-set enrichment analysis; HOMA-IR—Homeostasis-model assessment of insulin resistance; HOMA- β—Homeostasis-model assessment of insulin secretion; OXPHOS—Oxidative phosphorylation; QUICKI—Quantitative insulin-sensitivity-check index; qRT-PCR—Quantitative real-time PCR; SAT—Subcutaneous adipose tissue; T2D—Type 2 diabetes; TCA—Tricarboxylic acid
