# Supplementary material for: A Signature of Exaggerated Adipose Tissue Dysfunction in Type 2 Diabetes Is Linked to Low Plasma Adiponectin and Increased Transcriptional Activation of Proteasomal Degradation in Muscle
Source: Cells. 2022 Jun 23;11(13):2005. doi: 10.3390/cells11132005 (PMC9265693; doi:10.3390/cells11132005)
Supplement: Supplementary file 1 [file cells-11-02005-s001.zip › Supplementary Tables S1--S4 .pdf]

Supplementary Table S1

TaqMan assays for the selected genes validated with qRT-PCR.

Tissue	Gene	TaqMan assay
**Adipose tissue**		
	ACADS	Hs00916595_g1
	ADIPOQ	Hs00605917_m1
	ATP5B	Hs00969569_m1
	ATP5H	Hs01046889_gH
	CD68	Hs02836816_g1
	COL5A1	Hs00609140_m1
	COL6A2	Hs00242484_m1
	COX5A	Hs01561737_g1
	FH	Hs00895616_m1
	HADH	Hs00997450_m1
	LAMA2	Hs01124081_m1
	NDUFB8	Hs00428204_m1
	PPARGC1A	Hs01016719_m1
	SDHD	Hs00829723_g1
	UQCRC2	Hs00996395_m1
Reference gene	*ACTB*	Hs01060665_g1
**Skeletal muscle**		
	ACADVL	Hs00817723_g1
	ACVR2B	Hs00609603_m1
	ADIPOR1	Hs00360422_m1
	ADIPOR2	Hs00226105_m1
	ATP5A1	Hs00900735_m1
	COX5B	Hs00426948_m1
	CPT1B	Hs00992664_m1
	CS	Hs02574374_s1
	ETFA	Hs00164511_m1
	FBXO32	Hs01041408_m1
	FOXO3	Hs00818121_m1
	HADH	Hs00193428_m1
	MEF2C	Hs00231149_m1
	MSTN	Hs00976237_m1
	MYF5	Hs00271574_m1
	MYF6	Hs00231165_m1
	MYH1	Hs00428600_m1
	MYH2	Hs00430042_m1
	MYH7	Hs01110632_m1
	MYOD1	Hs00159528_m1

	MYOG	Hs01072232_m1
	MYOZ1	Hs00222007_m1
	NDUFS1	Hs00192297_m1
	PDK4	Hs01037712_m1
	PGAM2	Hs00165474_m1
	PPARGC1A	Hs01016719_m1
	PPP3CA	Hs00174223_m1
	PPP3CB	Hs00236113_m1
	PRKAA2	Hs00178903_m1
	PRKAG3	Hs00179660_m1
	PSMA2	Hs00746751_s1
	PSMB3	Hs00600803_m1
	PSMB6	Hs01572709_g1
	PSMD1	Hs00160631_m1
	SDHA	Hs00188166_m1
	SMAD7	Hs00998193_m1
	TRIM63	Hs00822397_m1
	UQCRC1	Hs00163415_m1
Reference gene	*IPO8*	Hs00183533_m1
Reference gene	*POLR2A*	Hs00172187_m1
Reference gene	*PPIA*	Hs00172187_m1

Supplementary Table S2

Anthropometric characteristics of males and females

	Obese controls	Type 2 diabetes	P-value
Males (n)	12	10	
BMI (kg/m^2)	30.5 ± 1.0	29.7 ± 1.4	0.66
Fat mass (kg)	29.9 ± 2.1	27.8 ± 2.7	0.55
Fat free mass (kg)	67.2 ± 2.5***	66.4 ± 2.2***	0.80
Percent fat mass (%)	30.7 ± 1.7***	28.9 ± 1.7***	0.47
Waist circumference (cm)	107 ± 3	109 ± 4	0.64
Hip circumference (cm)	106 ± 1	107 ± 33	0.84
Waist-hip ratio	1.01 ± 0.02	1.02 ± 0.02*	0.65
Females (n)	7	7	
BMI (kg/m^2)	30.6 ± 1.0	31.0 ± 1.8	0.85
Fat mass (kg)	34.0 ± 3.8	35.7 ± 3.6	0.76
Fat free mass (kg)	46.4 ± 1.6	47.4 ± 1.6	0.67
Percent fat mass (%)	41.6 ± 2.2	42.3 ± 1.9	0.82
Waist circumference (cm)	99 ± 4	97 ± 5	0.81
Hip circumference (cm)	105 ± 2	108 ± 8	0.77
Waist-hip ratio	0.94 ± 0.03	0.91 ± 0.04	0.68

Study participants were studied after an overnight fast. Data represent means ± SEM. *P<0.05, **P<0.01 and ***P<0.001 vs. females.

Supplementary Table S3

mRNA expression of genes involved in regulation of muscle mass and substrate metabolism

Gene	Obese controls	Type 2 diabetes	P-value
FOXO3	1.00 ± 0.09	0.77 ± 0.07	0.07
MSTN	1.00 ± 0.17	1.83 ± 0.20	0.005
ACVR2B	1.00 ± 0.06	1.19 ± 0.07	0.05
SMAD7	1.00 ± 0.08	0.95 ± 0.06	0.60
MYOD1	1.00 ± 0.09	0.78 ± 0.05	0.06
MYF5	1.00 ± 0.06	1.03 ± 0.08	0.73
MYF6	1.00 ± 0.08	1.08 ± 0.09	0.54
MYOG	1.00 ± 0.07	0.87 ± 0.05	0.12
MYH1	1.00 ± 0.33	1.82 ± 0.26	0.06
MYH2	1.00 ± 0.10	0.97 ± 0.07	0.82
MYH7	1.00 ± 0.11	0.96 ± 0.14	0.83
MYOZ1	1.00 ± 0.05	1.02 ± 0.03	0.72
MEF2C	1.00 ± 0.06	0.99 ± 0.04	0.87
PPP3CA	1.00 ± 0.04	1.26 ± 0.05	0.0004
PPP3CB	1.00 ± 0.04	1.09 ± 0.04	0.13
NDUFS1	1.00 ± 0.08	0.91 + 0.04	0.32
SDHA	1.00 ± 0.07	0.92 + 0.05	0.35
UQCRC1	1.00 ± 0.06	0.92 + 0.04	0.25
COX5B	1.00 ± 0.08	0.93 ± 0.03	0.45
ATP5A1	1.00 ± 0.07	0.94 ± 0.04	0.50
CS	1.00 ± 0.08	1.06 ± 0.04	0.54
CPT1B	1.00 ± 0.09	0.95 ± 0.05	0.59
ETFA	1.00 ± 0.07	1.02 ± 0.03	0.77
ACACDVL	1.00 ± 0.09	0.96 ± 0.08	0.71
HADH	1.00 ± 0.05	1.13 ± 0.05	0.08
PGAM2	1.00 ± 0.07	1.00 ± 0.05	0.98
ADIPOR1	1.00 ± 0.04	0.98 ± 0.03	0.73
ADIPOR2	1.00 ± 0.06	1.10 ± 0.05	0.36
PPKAA2	1.00 ± 0.05	0.99 ± 0.05	0.90
PRKAG3	1.00 ± 0.14	1.39 ± 0.13	0.06
PDK4	1.00 ± 0.24	0.88 ± 0.27	0.76
PPARGC1A	1.00 ± 0.09	0.89 ± 0.05	0.28

Muscle transcript levels by qRT-PCR of genes involved in the regulation of muscle mass and substrate metabolism. Data represent means ± SEM.

Supplementary Table S4

Expression of pro-inflammatory adipokines and putative myokines

Affy ID	Gene Symbol	Protein Name	Fold change	P-value	FDR
Pro-inflammatory adipokines					
209496_at	*RARRES2*	Chemerin	-1.21	0.02	0.44
203381_s_at	*APOE*	Apolipoprotein E	1.14	0.31	0.86
216598_s_at	*CCL2*	MCP-1	-1.11	0.73	0.96
220570_at	*RETN*	Resistin	1.07	0.39	0.87
205207_at	*IL6*	Interleukin 6	-1.06	0.47	0.89
203980_at	*FABP4*	Fatty acid binding protein 4	-1.06	0.00	0.17
217739_s_at	*PBEF1*	Visfatin	1.02	0.81	0.97
219140_s_at	*RBP4*	Retinol binding protein 4	-1.01	0.81	0.97
207092_at	*LEP*	Leptin	1.01	0.94	0.99
205382_s_at	*CFD*	Complement factor D (adipsin)	-1.00	0.94	0.99
Putative myokines					
207145_at	*GDF8*	Growth differentiation factor 8 (myostatin)	1.66	0.03	0.25
209101_at	*CTGF*	Connective tissue growth factor	-1.28	0.30	0.62
207433_at	*IL10*	Interleukin 10	-1.11	0.15	0.47
206382_s	*BDNF*	Brain-derived neurotrophic factor	-1.07	0.61	0.82
221576_at	*GDF15*	Growth differentiation factor 15	-1.07	0.31	0.63
204421_s_at	*FGF2*	Fibroblast growth factor 2 (basic)	-1.06	0.46	0.72
208782_at	*FSTL1*	Follistatin-like 1	-1.06	0.58	0.80
221009_s_at	*ANGPTL4*	Angiopoietin-like 4	-1.06	0.34	0.65
205207_at	*IL6*	Interleukin 6	1.05	0.47	0.73
221433_at	*FGF21*	Fibroblast growth factor 21	-1.05	0.39	0.68
205266_at	*LIF*	Leukemia inhibitory factor	1.04	0.53	0.77
201893_x_at	*DCN*	Decorin	1.04	0.48	0.74
205992_s_at	*IL15*	Interleukin 15	1.04	0.50	0.75
200665_s_at	*SPARC*	Secreted protein, acidic, cysteine-rich (osteonectin)	1.03	0.83	0.93
216546_s_at	*CHI3L1*	Chitinase 3-like 1 (YKL40)	-1.01	0.93	0.97

The microarray data set was explored to identify possible changes in expression of pro-inflammatory adipokines in SAT and putative myokines in muscle of patients with T2D compared with overweight/obese controls. In this table the probe sets with the highest expression are shown. P-values are unadjusted. An FDR<0.1 is considered significant.
